# Transmission dynamics of co-endemic *Plasmodium vivax* and *P*. *falciparum* in Ethiopia and prevalence of antimalarial resistant genotypes

**DOI:** 10.1371/journal.pntd.0005806

**Published:** 2017-07-26

**Authors:** Eugenia Lo, Elizabeth Hemming-Schroeder, Delenasaw Yewhalaw, Jennifer Nguyen, Estifanos Kebede, Endalew Zemene, Sisay Getachew, Kora Tushune, Daibin Zhong, Guofa Zhou, Beyene Petros, Guiyun Yan

**Affiliations:** 1 Department of Biological Sciences, University of North Carolina at Charlotte, Charlotte, North Carolina, United States of America; 2 Program in Public Health, University of California, Irvine, California, United States of America; 3 Department of Medical Laboratory Sciences and Pathology, College of Public Health and Medical Sciences, Jimma University, Jimma, Ethiopia; 4 College of Natural Sciences, Addis Ababa University, Addis Ababa, Ethiopia; 5 Department of Health Services Management, College of Public Health and Medical Sciences, Jimma University, Jimma, Ethiopia; University of Sao Paolo, BRAZIL

## Abstract

Ethiopia is one of the few African countries where *Plasmodium vivax* is co-endemic with *P*. *falciparum*. Malaria transmission is seasonal and transmission intensity varies mainly by landscape and climate. Although the recent emergence of drug resistant parasites presents a major issue to malaria control in Ethiopia, little is known about the transmission pathways of parasite species and prevalence of resistant markers. This study used microsatellites to determine population diversity and gene flow patterns of *P*. *falciparum* (*N* = 226) and *P*. *vivax* (*N* = 205), as well as prevalence of drug resistant markers to infer the impact of gene flow and existing malaria treatment regimes. *Plasmodium falciparum* indicated a higher rate of polyclonal infections than *P*. *vivax*. Both species revealed moderate genetic diversity and similar population structure. Populations in the northern highlands were closely related to the eastern Rift Valley, but slightly distinct from the southern basin area. Gene flow via human migrations between the northern and eastern populations were frequent and mostly bidirectional. Landscape genetic analyses indicated that environmental heterogeneity and geographical distance did not constrain parasite gene flow. This may partly explain similar patterns of resistant marker prevalence. In *P*. *falciparum*, a high prevalence of mutant alleles was detected in codons related to chloroquine (*pfcrt* and *pfmdr*1) and sulfadoxine-pyrimethamine (*pfdhps* and *pfdhfr*) resistance. Over 60% of the samples showed *pfmdr*1 duplications. Nevertheless, no mutation was detected in *pfK*13 that relates to artemisinin resistance. In *P*. *vivax*, while sequences of *pvcrt*-*o* were highly conserved and less than 5% of the samples showed *pvmdr* duplications, over 50% of the samples had *pvmdr*1 976F mutation. It remains to be tested if this mutation relates to chloroquine resistance. Monitoring the extent of malaria spread and markers of drug resistance is imperative to inform policy for evidence-based antimalarial choice and interventions. To effectively reduce malaria burden in Ethiopia, control efforts should focus on seasonal migrant populations.

## Author summary

Sub-Saharan Africa is home to nearly 90% of malaria cases. In Ethiopia, two-thirds of the population lives in areas at risk of malaria infection. Malaria spread via human migrations and emergence of drug resistant parasites are major issues to malaria control in this country. Our study used microsatellite markers to determine gene flow patterns of *P*. *falciparum* and *P*. *vivax* in different parts of Ethiopia. We found that gene flow occurred across broad geographical distance and that environmental heterogeneity did not appear to influence gene flow. Unconstrained parasite gene flow may partly explain similar patterns of resistance marker prevalence across the country. While no mutation was detected in *pfK*13 that relates to artemisinin resistance in *P*. *falciparum*, over 50% of our *P*. *vivax* samples had *pvmdr*1 976F mutation that may relate to chloroquine resistance. This merits further clinical observations and/or *in vitro* testing. Our findings heighten the concern of chloroquine resistance for *P*. *vivax* malaria after more than a decade-application and suggests alternative treatment regime to alleviate the problem. Broadly, malaria control efforts should focus on seasonal migrant populations to effectively reduce malaria burden in Ethiopia.

## Introduction

Despite considerable progress towards malaria control, two-thirds of the population in Ethiopia, i.e., approximately 66 million people, reside in areas of low or high malaria transmission [1]. Apart from human factors such as population mobility, urbanization, and agricultural development, emergence of drug resistant parasites and insecticide resistance present a major hurdle to malaria control programs in Ethiopia and worldwide [2]. Reports of emerging *Plasmodium vivax* resistance to chloroquine (CQ) in Ethiopia threaten the efficacy of *P*. *vivax* treatment [3–6]. Also, the well-documented emergence of *P*. *falciparum* resistance to artemisinin in Southeast Asia may endanger current malaria treatment programs in Ethiopia, given that both CQ and sulfadoxine-pyrimethamine (SP) resistance originated in Southeast Asia and spread quickly to East Africa [7]. Thus, knowing how malaria parasites spread as well as monitoring prevalence of drug resistant markers in high-risk areas are important to informing antimalarial interventions.

While *P*. *vivax* is the most widespread human malaria parasite, it is rare in Africa where *P*. *falciparum* predominates. Due to its low prevalence in the continent, little is known about the transmission patterns of *P*. *vivax* in Africa. Ethiopia is unique in that *P*. *vivax* is co-endemic with *P*. *falciparum* at approximately equal case incidence rates. Other African countries with significant *P*. *vivax* infections are Eritrea, Sudan, and Madagascar [1]. Although Ethiopia carries a substantial malaria burden, information on the transmission dynamics and spread of drug resistance across the country is scarce.

*P*. *vivax* and *P*. *falciparum* exhibit different biological and epidemiological features. Compared to *P*. *falciparum*, *P*. *vivax* has a broader temperature tolerance, an early onset of gametocyte development, and a dormant life cycle stage, the hypnozoite, in the host liver that can cause relapse. Relapse infections may present opportunities for *P*. *vivax* to exchange and disseminate alleles at any time of the year rather than only the transmission season [8]. Population genetic diversity and structure are thus expected to be different between *P*. *vivax* and *P*. *falciparum* even when the two species coexist. For instance, in Cambodia [9], the Indo-West Pacific [10–12], and the Brazilian Amazonia [13], *P*. *vivax* revealed a higher microsatellite diversity than its sympatric *P*. *falciparum*. A similar contrast was observed in Papua New Guinea where *P*. *vivax* showed a higher AMA1 gene diversity than *P*. *falciparum* [14]. Globally, both *P*. *falciparum* and *P*. *vivax* in Africa were markedly differentiated from those in Southeast Asia and Oceania [15,16], reflecting a clear continental disjunction. While *P*. *vivax* is genetically most diverse in Southeast Asia [17,18], *P*. *falciparum* diversity is the highest in East and West Africa compared to Southeast Asia and Oceania [19,20]. Such differences could be tightly associated with the historical levels of transmission intensity. Comparing genetic diversity and structure between the two species at the same endemic setting would shed light on the biological relevance on malaria epidemiology.

Apart from transmission dynamics, the biological differences between *P*. *falciparum* and *P*. *vivax* have added a layer of complexity to antimalarial treatment programs in Ethiopia. First-line treatment for *P*. *falciparum* is arthemether-lumefantrine (AL), which replaced SP in 2005 due to increasing and widespread SP resistance. While CQ was withdrawn in 1998 due to a high prevalence of CQ resistance in *P*. *falciparum*, it remains the first-line treatment for *P*. *vivax* in Ethiopia [2]. Genetic markers for CQ (*pfcrt*T76), SP (*pfdhfr*I51-R59-N108 + *pfdhps*G437-E540), and artemisinin (*Kelch13*-propeller region) resistance in *P*. *falciparum* have been well documented [21–24]. For *P*. *vivax*, although there is no clear evidence that variants in *pvcrt-o* and *pvmdr*1 are associated with CQ resistance, mutations including T958M, Y976F and F1076L in *pvmdr*1, as well as a K-10 insertion (lysine (K) insertion on chromosome 10) in *pvcrt-o* have been suggested as possible genetic markers [25,26]. Mutation frequency of these genes largely depends on the level of drug usage and the extent of the spread of resistant genotypes. For instance, the *pfcrt*T76 mutation frequency was almost 100% among clinical and asymptomatic *P*. *falciparum* samples from 2004–2012 in south-central Ethiopia [27–29]. In the case of mixed infections with the two species where only *P*. *vivax* diagnosed, *P*. *falciparum* is still exposed to CQ despite the change of *P*. *falciparum* first-line treatment more than a decade ago. On the contrary, the frequency of *pfdhfr* and *pfdhps* quintuple mutations had decreased significantly from 2005 to 2008 since the withdrawal of SP in 2005 [30,31], indicative of relaxed selection in the *P*. *falciparum* populations. Mutations in the kelch (*K13*)-propeller region are markers of artemisinin resistance [24]. Resistance-associated *pfK13* mutations are widely prevalent in Southeast Asia but those mutations have not yet been common in Africa [32]. Recently, a new nonsynonymous mutation at *pfK13* position 579 (M579I) was detected in a *P*. *falciparum* strain that was indigenous to Equatorial Guinea and shown to be artemisinin resistant based on *in vitro* testing [33]. Careful surveillance of *pfK13*-propeller region mutations in Ethiopia will be especially useful to detecting the spread of artemisinin resistance from Southeast Asia to Africa.

This study examined and compared population diversity and gene flow patterns between *P*. *falciparum* and *P*. *vivax* in the northern, eastern, and southern parts of Ethiopia with low to moderate level of malaria transmission. Specifically, we investigated if landscape heterogeneity impacts parasite gene flow by testing the association between landscape factors and population genetic structure. We tested three competing hypotheses of factors that may influence gene flow: 1) factors related to vector ecology (land cover and precipitation); 2) factors related to human movement (distance to roads); and 3) factors related to environment (elevation, which is tightly correlated with temperature). Further, we inferred how gene flow pattern relates to the prevalence of drug resistance markers. This knowledge will help inform how malaria parasites and drug resistance spread, how *P*. *vivax* and *P*. *falciparum* epidemiology influences genetic structures, as well as antimalarial drug efficacy in Ethiopia. Monitoring for markers of antimalarial drug resistance is imperative to informing public health interventions.

## Materials and methods

### Ethics statement

Scientific and ethical clearance was obtained from the institutional scientific and ethical review boards of Jimma and Addis Ababa Universities in Ethiopia and University of California, Irvine, USA. Written informed consent/assent for study participation was obtained from all consenting heads of households, parents/guardians (for minors under age of 18), and each individual who was willing to participate in the study.

### Study areas and sample collection

**Fig 1:**
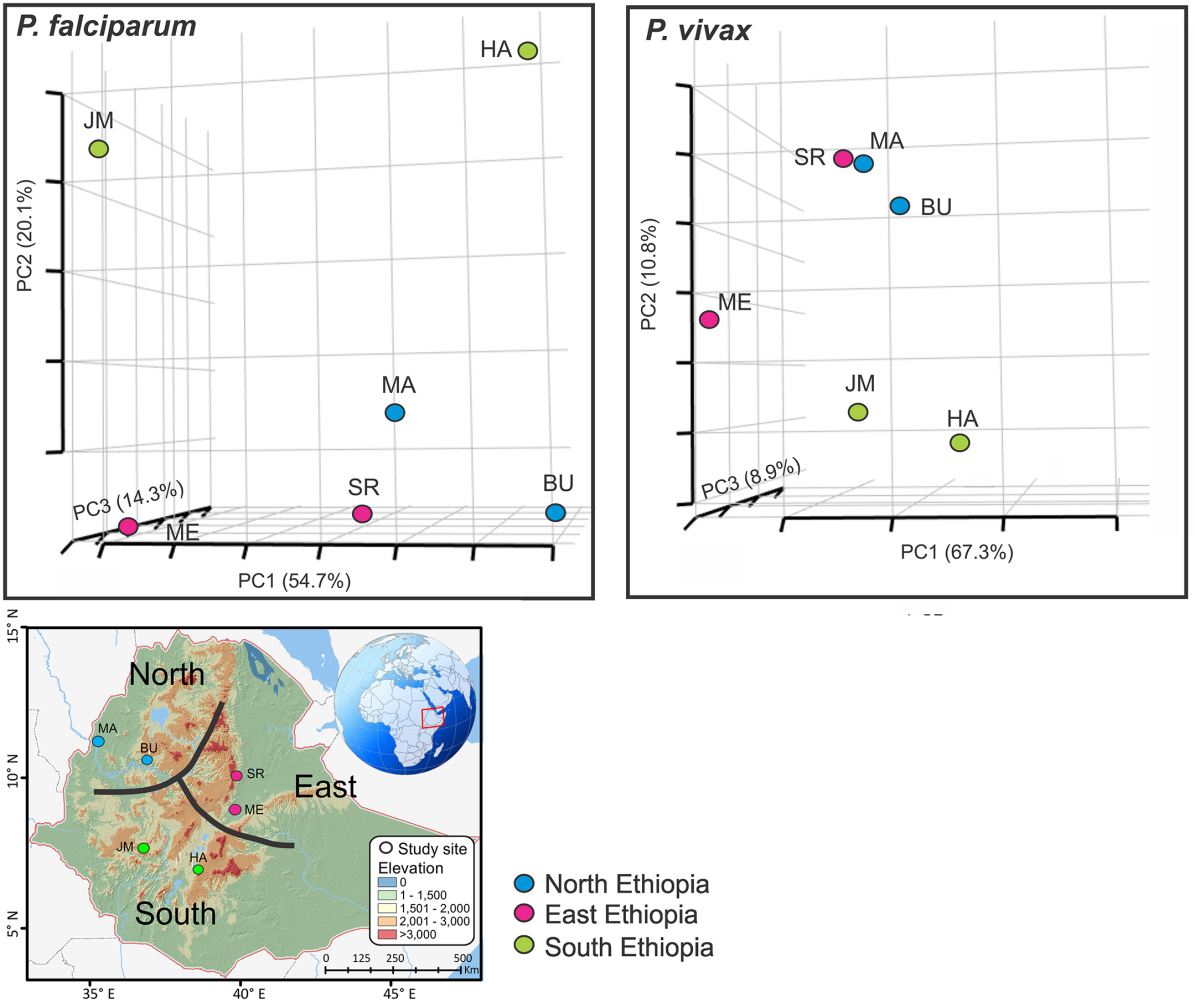
Three dimensional scatter plots of pairwise *D*_S_ values (an analog of *F*_ST_) showing the genetic relatedness of *Plasmodium falciparum* and *P*. *vivax* among sites. The first three axes that contain nearly 90% of the total variation are shown. Locations of the studied sites from different parts of Ethiopia are presented in the map below as well as S1 Table.

Clinical samples from six study sites representing the northern highland (MA: Mankush and BU: Bure), eastern Rift Valley (SR: Shewa Robit and ME: Metehara), and southern basin area (JM: Jimma and HA: Halaba) of Ethiopia were collected during the peak transmission season (September-November) of 2014 (Fig 1; S1 Table). This area encompasses an elevation gradient from ca. 50m in the basin to over 2,500m in the highlands west of the Great Rift Valley. Finger-prick blood samples were collected from malaria symptomatic (who has fever with axillary body temperature > 37.5°C and with confirmed asexual stages of malaria parasite based on microscopy) or febrile patients visiting the health centers or hospitals at each of the study sites. Thick and thin blood smears were prepared for microscopic examination and three to four spots of blood, equivalent to ~50 μl, from each individual were blotted on Whatman 3MM filter paper. Parasite DNA was extracted from dried blood spots by the Saponin/Chelex method [34]. Nested and quantitative PCR were performed to identify and confirm parasite species of the infected samples [35]. A total of 226 and 205*P*. *falciparum* and *P*. *vivax* samples (ranged from 18–58 samples per site) were included in microsatellite analyses.

### Microsatellite genotyping

Thirteen single-copy microsatellites with tri- or tetranucleotide repeats, which mapped to 14 chromosomes, were typed for *P*. *falciparum* and *P*. *vivax*, respectively (S2 Table). Alleles were PCR-amplified with the published oligonucleotide primers [36–38]. For each PCR reaction, 2 μl of genomic DNA were used with 2 mM MgCl_2_, 2 μM of each primer (all forward primers were labeled with fluorescent dyes; Applied Biosystems, Foster City, CA), and 10μl of 2×DreamTaq Green PCR Master Mix (Thermo Scientific, Waltham, MA) in a final volume of 20 μl. PCR cycling conditions were as follows: 2 min, 94°C; (30 sec, 94°C; 40 sec, 58°C; 50 sec, 72°C) for 40 cycles; 5 min, 72°C. After PCR amplification, products were pooled into four groups based on size differences: TAA87+PFPK2+POLY2+9735, TAA42+TAA81+TAA109, PE87a+PFG377+POLYα, TAA60+TA80+TA116 for *P*. *falciparum;* MS1+MS3+MS4+MS5, MS8+MS9+MS16, MS10+MS12+MS15, MS20+Pv1.501+Pv3.27 for *P*. *vivax* (S2 Table). The pooled products were separated on an ABI 3730 sequencer and all allele sizes were determined and visualized in Peak Scanner. To avoid background signal and potential artifacts, a threshold of 500 relative fluorescent units was set for peak detection. For each sample, the dominant allele and any alleles with a minimum of 33% height of the dominant allele were scored [36].

### Data analyses

#### Linkage disequilibrium and genetic diversity

All analyses were performed separately on the *P*. *falciparum* and *P*. *vivax* datasets. To examine whether the microsatellite loci represent an independent set of markers of the parasite genome, linkage disequilibrium (LD) was tested by Fisher’s exact test for each pair of loci with GenePop version 4.2, using the Markov chain method with 100 batches and 10,000 iterations per batch [39]. Significance values were adjusted by sequential Bonferroni correction for multiple comparisons. In addition, mulltilocus LD was assessed among the parasite samples for each of the populations using the web-based LIAN 3.5 [40]. The standardized index of association (*I*_A_^S^), which measures the strength of linkage disequilibrium and views as a function of the rate of recombination among samples, was calculated with 10,000 random permutations of the data.

The percentage of polyclonal infections (i.e., samples with more than one allele at any given locus) as well as multiplicity of infections (MOI: the number of genetically distinct clones present within a host) were estimated for each of the study sites, respectively, for *P*. *falciparum* and *P*. *vivax*. For each sample, MOI was scored as the maximum number of alleles observed when all loci were taken into account and the average MOI was calculated for each population.

Genotypic variation was calculated in GenoDive version 2.0b27 [41]. We first calculated genetic distances using the method of Smouse and Peakall, a squared Euclidean distance based on the number of times a certain allele was found in the two individuals [42]. The minimal distance class was set as threshold to identify the following: (i) the number of multilocus genotypes (G); (ii) Simpson’s diversity index (D), also known as Nei’s genetic diversity corrected for sample size that ranges from zero (where two randomly chosen individuals in a population share a single genotype) to one (where individuals have different genotypes); and (iii) genotype evenness (E) that ranges from zero (where one or a few genotypes dominate in a population) to one (where all genotypes are of equal frequency in a population). In addition, the number of effective alleles and expected heterozygosity were estimated for each study site.

### Population structure and isolation-by-distance

A model-based Bayesian method implemented in STRUCTURE v2.3.4 was performed to examine partitioning of individuals to genetic clusters [43]. The number of clusters (*K*) was determined by simulating a range of *K* values from 1 (no genetic differentiation among all sites) to 6 (all sites were genetically differentiated from one another). The posterior probability of each value was then used to detect the modal value of Δ*K*, a quantity related to the second order rate of change with respect to *K* of the likelihood function [44]. Posterior probability values were estimated using a Markov Chain Monte Carlo (MCMC) method. A burn-in period of 500,000 iterations followed by 10^6^ iterations of each chain was performed to ensure convergence of the MCMC. Each MCMC chain for each value of *K* was run ten times with the ‘independent allele frequency’ option that allows individuals with ancestries in more than one group to be assigned into one cluster. Individuals were assigned into *K* clusters according to membership coefficient values (Q) ranged from 0 (lowest affinity to a cluster) to 1 (highest affinity to a cluster). The partitioning of clusters was visualized with DISTRUCT [45]. Neighboring-joining trees were constructed using T-REX [46,47] to show the genetic relatedness among *P*. *falcipar*um and *P*. *vivax* samples. The squared Euclidean distance, which is based on the number of times a certain allele found in two individuals [48], was used for tree constructions. The resulted trees were visualized in FigTree v1.4.2.

An *F*_ST_ analysis was conducted using θ, an *F*_ST_-estimator in SPAGeDi v1.2e [49]. *F*_ST_ values were tested for significance using 10,000 permutations. Genetic differentiation among sites was displayed by multidimensional scaling plot based on the estimated *D*_S_ values (an analog of *F*_ST_) in R v3.3.0. Furthermore, an analysis of molecular variance (AMOVA) was used to determine the hierarchical distribution of genetic variance within and among populations, as well as among regions (north, east, and south of Ethiopia) using GENALEX [50]. The relationships between genetic distances (*D*_S_ values) and Euclidean geographical distance (estimated from spatial coordinates using R for multivariate and spatial analysis; [51]) were examined by Mantel tests (10,000 randomizations) and reduced major axis (RMA) regression in the Isolation By Distance v3.23 [52].

### Bottlenecks and migration rates

Signature of genetic bottleneck was detected with BOTTLENECK v1.2.02 [53]. Only sites with a sample size of 20 or above were included for statistical significance. Two tests were performed using three different mutation models: the infinite alleles model (IAM), the stepwise mutation model (SMM), and a combination of those two extreme hypotheses, the two-phase model (TPM). First was the overall distribution of allele frequency classes. Second was the Wilcoxon-signed rank test to compare the number of loci that present a heterozygosity excess to the number of such loci expected by chance only.

Frequency of gene flow among populations was estimated for each parasite species by a maximum-likelihood analysis implemented in Migrate-N v2.4.4 [54]. Parameters including Θ (defined as 4*N*_e_μ, where *N*_e_ is the effective population size and μ is the mutation rate per generation and site) and *M* (m/μ, where m is the immigration rate scaled by mutation rate) were estimated. Four independent runs were conducted with the Brownian motion model using 10 short chains with 5,000 sampled genealogies and three long chains with 50,000 sampled genealogies to obtain the mean and range of Θ and *M* values. In addition, we inferred migration rate using a Bayesian approach implemented in BayesAss v3 [55], which is not dependant on the assumption of equilibrium and can be used with populations that are not in migration-drift or Hardy-Weinberg equilibrium. A MCMC algorithm was used to estimate the posterior probability distribution of the proportion of migrants from one population to another. We performed the analyses with 9×10^6^ iterations, with a burn-in of 10^6^ iterations, and a sampling frequency of 2,000 to ensure the parameters of the model were converged. The correlation between migration rate and geographical distance was tested for all pairs of populations.

### Landscape genetics

To test for the effects of landscape factors on gene flow between populations, we performed a landscape genetic analysis as follows. First, we created landscape resistance surfaces based on our predictions of the factors influencing gene flow of *Plasmodium* species, specifically factors influencing vector ecology (land cover and precipitation), human movement (distance to roads), and *Plasmodium* biology (elevation, which is tightly correlated with temperature). A resistance surface is a spatial layer in which each cell in a grid is assigned a value that represents the degree to which that cell constrains gene flow or movement [56]. These values were often based on numerous assumptions about relationships between a landscape or environmental feature and the ability of a given organism to move through that feature. Landscape resistance surfaces were derived from publicly available data: NASA MODIS MCD12Q2 for land cover (forest, shrubland, woody savanna, savanna, grassland, cropland, and sparsely vegetated) [57,58]; NASA SRTM v4.1 for elevation [59]; WorldClim v1.4 for precipitation [60]; and Roads Africa shapefile in ArcGIS for distance to roads. All raster files were resampled to a resolution of 1km in ArcGIS 10. Next, we used ResistanceGA to optimize landscape resistance surfaces based on our genetic data [61]. ResistanceGA uses a genetic algorithm to unbiasedly assign landscape resistance values to continuous or categorical data. Circuitscape v.4.05 was used to measure resistance distance between populations [62]. Circuitscape relies on electrical circuit theory to predict landscape connectivity and incorporates all possible pathways between populations into the resistance distance measure. To test the fit of resistance surfaces in relation to the genetic data, linear mixed effects models with the maximum likelihood population effects (MLPE) were fit in *lme4* [63]. Finally, Akaike information criterion with a penalty for extra parameters (AICc) was calculated from the linear mixed effect model and used as the means for model selection.

### Resistance gene sequencing

Five gene regions that are putatively associated with CQ (*pfcrt* and *pfmdr*1), SP (*pfdhps* and *pfdhfr*), and artemisinin (*pf*K13) resistance were sequenced with *P*. *falciparum* samples. Polymorphisms were examined for the following codons: *pfcrt*–codon76; *pfmdr1*–codons 86, 184, 1042, and 1246; *pfdhps*–codons 396, 436, 437; *pfdhfr*–codons 51, 59, 108; *pf*K13–codons 476, 493, 519, 532, 539, 543, 578, 579, 580, 582, and 590. In addition, two gene regions that are putatively associated with CQ resistance (*pvcrt*-o and *pvmdr*1) were sequenced with *P*. *vivax* samples. Polymorphisms were examined for the following: *pvcrt-o*–a (AAG) insertion at codon 10 (K10 insert), codon 117; pvmdr*1*–codons 958, 976, and 1076. Amplification was conducted in a 20μl reaction mixture containing 3μl of genomic DNA, 12.5μl of 2×DreamTaq Green PCR Master Mix, and 10 nmol of forward and reverse primers based on the published protocols [21–23,32,64]. PCR products were then purified the by the SAP-ExoI method (Affymetrix, Santa Clara, CA) and sequenced in both directions by Sanger sequencing (GENEWIZ).

### *Pfmdr*1 and *Pvmdr*1 gene copy estimation

The *pfmdr*1 gene copy number of *P*. *falciparum* was assessed by real-time PCR. Genomic DNA of *P*. *falciparum* clones 3D7 (which has a single copy of *pfmdr*1) was used as a calibrator and *pfβ-tubulin*, a house-keeping gene, was used as an internal control. The primers for *pfmdr*1 and *β-tubulin* were described previously [65]. For *P*. *vivax*, the Salvador I strain was used as a calibrator and the *pvaldolase* gene, which is known to be a single copy gene in *P*. *vivax*, was used as an internal control using the published primers [66].

Amplification was performed in triplicate in a total volume of 20 μl containing 10μl of SYBR Green PCR Master Mix, 0.75 μl of each of the sense and anti-sense primers (10 μM), 20 ng of genomic DNA and 3.5 μl of water. Reaction was performed in CFX96 Touch^™^ Real-Time PCR Detection System (Bio-Rad), with an initial denaturation at 95°C for 3 min, followed by 45 cycles at 94°C for 30 sec, 55°C for 30 sec, and 68°C for 1 min with a final 95°C for 10 sec. This was then followed by a melting curve step of temperature ranged from 65°C to 95°C with 0.5°C increment to determine the melting temperature of each amplified product. A negative control with no template was used in each run. Each sample was run in triplicates and the *C*_*t*_ values and melting temperature were recorded at the end of the reactions. The average and standard deviation of the three *C*_*t*_ values were calculated, and the average value was accepted if the standard deviation was lower than 0.32. The 2^ΔΔCt*±*SD^ method for relative quantification was used to estimate the gene copy number [66] and the results were expressed as the *N*-fold copy number of the targeted gene in relative to the reference. Fisher’s exact test (given small sample size) was used to test for significant differences in mutation prevalence and gene copy number among the study populations. All statistical analyses were performed in R (R Core Team 2013).

## Results

### Linkage disequilibrium and complexity of infections

No significant LD was detected for all pairwise combinations of microsatellite loci among the *P*. *falciparum* and *P*. *vivax* samples (Bonferroni corrected *P*>0.05). However, when all locus were pooled together in the analyses, *P*. *falciparum* in general showed a higher level of linkage and/or rate of recombination (*I*_A_^S^ values ranged from 0.005 in Bure (BU) to *I*_A_^S^ = 0.13 in Halaba (HA); all sites *I*_A_^S^ = 0.03, *P*<0.05 except BU) than *P*. *vivax* (*I*_A_^S^ values ranged from 0.003 in Mankush (MA) and Jimma (JM) to 0.02 in Shewa Robit (SR); all sites *I*_A_^S^ = 0.001, *P*>0.05; Table 1).

**Table 1:** Linkage disequilibrium and complexity of infection among *P*. *falciparum* and *P*. *vivax* samples by study sites. ‘ns’ denotes non-significant; ‘*’ denotes *P*<0.05.

	Site	*I*_A_^S^	# of polyclonal/total infections (%)	Mean MOI, median (range)
*P*. *falciparum*				
	BU	0.005^ns^	3/42 (7.1%)	1.09, 1 (1–3)
	MA	0.018*	4/36 (11.1%)	1.14, 1 (1–3)
	ME	0.055*	3/46 (6.5%)	1.07, 1 (1–2)
	SR	0.051*	1/33 (3.0%)	1.03, 1 (1–2)
	HA	0.126*	3/18 (16.7%)	1.17, 1 (1–2)
	JM	0.033*	6/51 (11.8%)	1.14, 1 (1–3)
	**All sites**	0.030*	**20/226 (8.8%)**	**1.10, 1 (1–3)**
*P*. *vivax*				
	BU	0.005^ns^	2/39 (5.1%)	1.03, 1 (1–2)
	MA	0.004^ns^	1/19 (5.3%)	1.05, 1 (1–2)
	ME	0.017^ns^	1/21 (4.8%)	1.05, 1 (1–2)
	SR	0.025^ns^	0/21 (0%)	1, 1 (1)
	HA	0.008^ns^	2/47 (4.3%)	1.04, 1 (1–2)
	JM	0.004^ns^	3/58 (5.2%)	1.07, 1 (1–3)
	**All sites**	**0.001**^**ns**^	**9/205 (4.4%)**	**1.04, 1 (1–3)**

Compared to *P*. *falciparum* (8.8%; 20/226; Table 1), *P*. *vivax* indicated a lower rate of polyclonal infections (4.3%; 9/205). Polyclonal samples were observed in all sites for *P*. *falciparum*, with the highest rate of polyclonal infections in the southern lowlands (HA: 16.7% and JM: 11.8%). For *P*. *vivax*, polyclonal infections ranged from 5.3% in Bure (BU) to 0% in Shewa Robit (SR), despite a slightly smaller sample size. Likewise, MOI for *P*. *falciparum* from all sites (mean MOI = 1.10; Table 1) was significantly higher than that of *P*. *vivax*, (mean MOI = 1.04, *P*<0.01), indicative of a higher complexity within *P*. *falciparum* infections.

Among all the polyclonal infections, 18 were bi-clonal of which two equally dominant alleles were detected in a single locus. We separated the genotypes of the two strains and included them in the analyses. For the 11 samples that showed >1 alleles in two or more loci, we were unable to confidently differentiate the genotypes of the different strains and thus these samples were discarded in the analyses (S3 Table).

### Genetic diversity comparison

Both *P*. *falciparum* and *P*. *vivax* revealed similar levels of allelic and genotypic diversity (Table 2). Nevertheless, genotypic evenness in *P*. *vivax* from the highlands (*E* = 0.75; BU and MA) was significantly lower than the other samples (*P*<0.01; two-tailed *t*-test; Table 2). This suggested a less even distribution of genotypes in these populations and that some genotypes were more common than the others. AMOVA indicated that most of the genetic variation was within populations in both *P*. *falciparum* and *P*. *vivax* (>84%; S4 Table). In *P*. *falciparum*, a greater proportion of variation was found among regions (13% among the north, east and south Ethiopia) than among populations in a region (3%); whereas in *P*. *vivax*, the proportion of variation among regions (8%) and populations (7%) were comparable and significant.

**Table 2:** Comparison of *Plasmodium falciparum* and *P*. *vivax* genetic diversity measures based on microsatellite markers.

Species; sites	Sample size	Genotypic diversity			Gene diversity	
		*G*	*D*	*E*	*N*_e_	*H*_e_
***P*. *falciparum***						
North Ethiopia						
Bure	42	36.75	0.97	0.94	2.99	0.59
Mankush	36	32.55	0.97	1	4.17	0.71
East Ethiopia						
Metehara	46	44.08	0.98	0.98	3.04	0.56
Shewa Robit	33	29.43	0.97	0.95	3.05	0.57
South Ethiopia						
Halaba	18	10.11	0.83	1	2.13	0.52
Jimma	51	47.29	0.98	0.96	3.60	0.66
**Total**	**226**	**198**	**0.95**	**0.97**	**2.81 (±0.34)**	**0.66 (±0.05)**
***P*. *vivax***						
North Ethiopia						
Bure	39	24.14	0.96	0.75	4.93	0.73
Mankush	19	12.08	0.67	0.75	2.46	0.66
East Ethiopia						
Metehara	21	14.02	0.88	0.72	3.64	0.69
Shewa Robit	21	19.31	0.89	0.93	3.07	0.72
South Ethiopia						
Halaba	47	40	0.98	0.91	3.78	0.59
Jimma	58	35.53	0.96	0.92	5.56	0.77
**Total**	**205**	**144**	**0.91**	**0.81**	**3.16 (±0.40)**	**0.69 (±0.06)**

**G**: Number of multilocus genotypes corrected for sample size

**D**: Simpson's diversity index corrected for sample size

**E**: Genotypic evenness;

**N**_**e**_: Number of effective alleles (Nielsen et al. 2003);

**H**_**e**_: Expected heterozygosity corrected for sample size (Nei 1978)

### Genetic clustering of samples

Populations in north and east Ethiopia were slightly differentiated from those in the south (Fig 1). This pattern was shown in both *P*. *falciparum* and *P*. *vivax*, though populations were more scattered in *P*. *falciparum*. The two northern populations (BU and MA) were genetically close to samples in the eastern Rift Valley (SR and ME; Fig 1).

**Fig 2:**
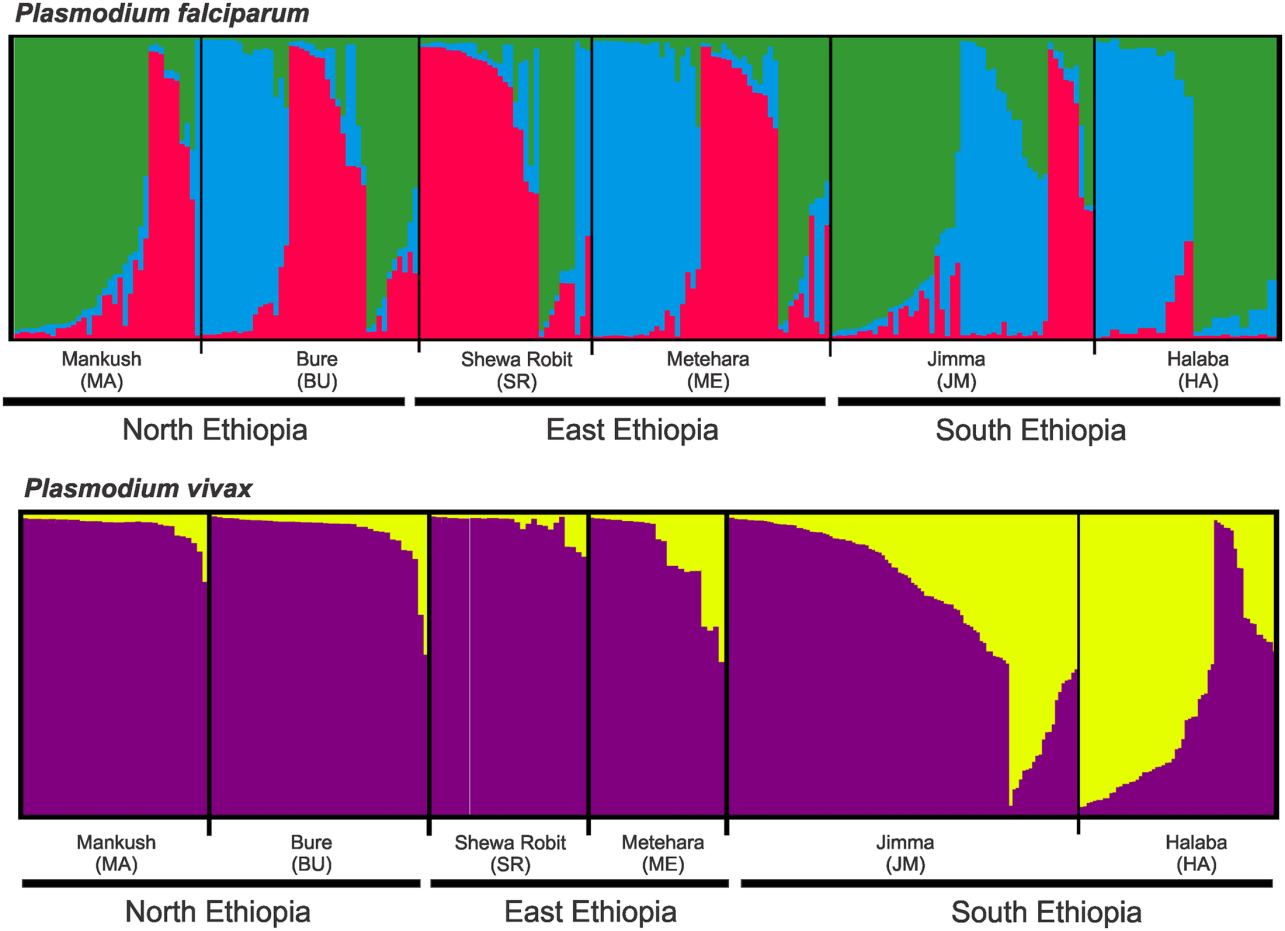
Bayesian inferences of the *K* clusters estimated by STRUCTURE among *Plasmodium falciparum* and *P*. *vivax* samples. The most probable clusters are labeled by different colors, and individuals are represented as columns. Within each column (individual), the extent of the component colors indicates the magnitude of the membership coefficient (Q) corresponding to each cluster. Q values of respective clusters are presented in S5 Table.

In *P*. *falciparum*, three most probable genetic clusters were detected by STRUCTURE analyses (Fig 2), but these clusters did not clearly represent geographical regions. The red cluster was most apparent in the northern (MA and BU) and eastern (SR and ME) populations, but less significant in the southern populations (JM and HA). All three genetic clusters were found in sites BU and ME, but the blue cluster was almost absent in MA and SR (Fig 2). In *P*. *vivax*, samples from north and east Ethiopia constituted predominantly the purple cluster, contrast with those from the south that constituted an admixture of the purple and yellow clusters. Unlike *P*. *falciparum*, the genetic composition between the northern and eastern populations was largely similar.

**Fig 3:**
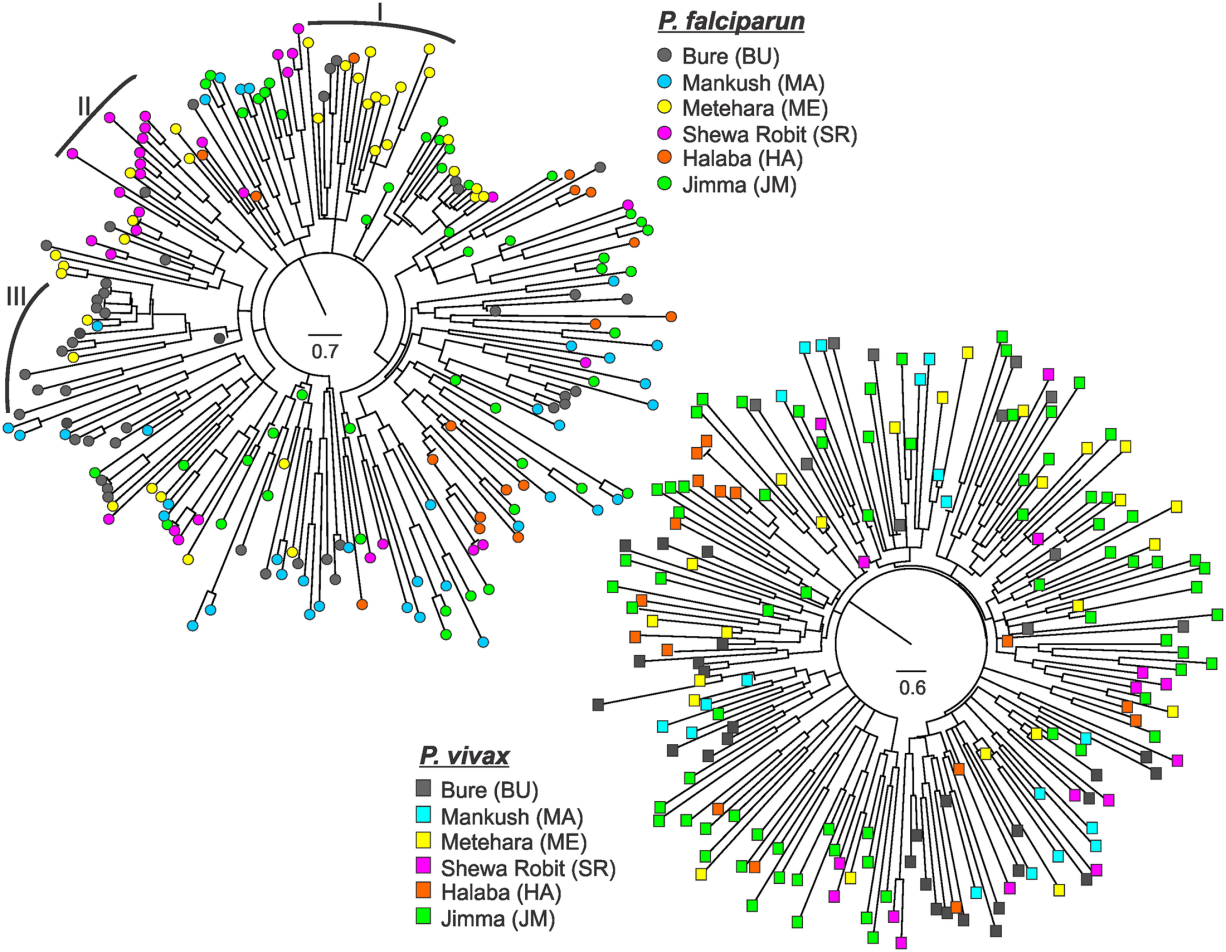
Neighbor-joining trees showing the genetic relatedness among *Plasmodium falciparum* and *P*. *vivax* samples. (A) Genetic relatedness among *P*. *falciparum* samples from the six study sites, shown by different color circle. Subclade-I represents a genetic cluster of samples predominantly from Metehara that were closely related; subclade-II represents a cluster of samples predominantly from Shewa Robit; and subclade-III represents a cluster of samples predominantly from Bure. (B) Genetic relatedness among *P*. *vivax* samples from the six study sites, shown by different color square.

Neighbor-joining trees did not indicate clear distinction among the *P*. *falciparum* and *P*. *vivax* population samples (Fig 3). Both trees had relatively short internodes but long terminal branches, which suggested that the parasite lineages were rapidly diverged from one another and that frequent gene exchange occurred among the populations. In *P*. *falciparum*, there were a number of subclades where parasites from the same population were genetically closely related (e.g., subclades I, II, and III in Fig 3A). However, in *P*. *vivax*, samples from different sites were clustered together in the same clade without clear differentiation (Fig 3B).

### Distance and landscape factors

Mantel tests indicated no significant association between geographical and genetic distances among populations of *P*. *falciparum* (*R*^2^ = 0.14, *P*>0.05) and *P*. *vivax* (*R*^2^ = 0.09, *P*>0.05), respectively. These results suggest that parasite gene flow was not limited by geographical distance. Further, for both *P*. *falciparum* and *P*. *vivax*, we found that none of the tested landscape factors explained pairwise genetic distance (*F*_ST_) among populations more than the Euclidean distance alone based on AICc (Table 3). These results indicated that the differences in land cover, elevation, precipitation, and distance to roads (a proxy for accessibility) did not significantly influence parasite gene flow and that our study populations were clearly connected (S1 Fig).

**Table 3:** Model fitness of resistance surfaces to pairwise *F*_ST_ values calculated using ResistanceGA. AICc: corrected Akaike Information Criterion; ω: Akaike weight.

Species	Landscape surface	AICc	ΔAICc	ω
***P*. *falcipaum***				
	Euclidean distance	-50.21	0	0.36
	Distance to roads	-49.90	0.31	0.32
	Elevation	-48.99	1.22	0.19
	Precipitation	-48.22	1.99	0.13
	Land cover	-15.78	34.43	0
***P*. *vivax***				
	Euclidean distance	-43.26	0	0.41
	Precipitation	-42.05	1.21	0.22
	Elevation	-41.95	1.32	0.21
	Distance to roads	-41.35	1.91	0.16
	Land cover	-7.13	36.14	0

### Demographic change and migrations

All populations of *P*. *falciparum* showed a normal L-shape distribution in allele frequency (Table 4), suggesting that these populations did not experience a recent severe bottleneck. In *P*. *vivax*, allele frequency was shown with a shifted mode in site SR (east Ethiopia), indicative of a significant genetic bottleneck. In addition, a significant excess of heterozygosity was observed in this population as well as other northern and eastern populations (ME and BU) under the IAM and SMM mutation models, suggestive of a deviation in the mutation-drift equilibrium.

**Table 4:** Summary of the parameters and results based on BOTTLENECK analyses.

Species	Site; sample size	Mode shift	Mutation model	Heterozygote excess
***P*. *falciparum***				
	North Ethiopia			
	Bure (n = 42)			
		Normal L-shaped	IAM	*P* = 0.11
			SMM	0.02*
			TPM	0.50
	Mankush (n = 36)			
		Normal L-shaped	IAM	0.05
			SMM	0.12
			TPM	0.05
	East Ethiopia			
	Metehara (n = 46)			
		Normal L-shaped	IAM	0.52
			SMM	0.002**
			TPM	0.13
	Shewa Robit (n = 33)			
		Normal L-shaped	IAM	0.25
			SMM	0.26
			TPM	0.50
	South Ethiopia			
	Jimma (n = 51)			
		Normal L-shaped	IAM	0.54
			SMM	0.25
			TPM	0.21
***P*. *vivax***				
	North Ethiopia			
	Bure (n = 39)			
		Normal L-shaped	IAM	0.56
			SMM	0.01*
			TPM	0.55
	East Ethiopia			
	Metehara (n = 21)			
		Normal L-shaped	IAM	0.02*
			SMM	0.41
			TPM	0.21
	Shewa Robit (n = 21)			
		Shifted	IAM	0.03*
			SMM	0.35
			TPM	0.12
	South Ethiopia			
	Halaba (n = 47)			
		Normal L-shaped	IAM	0.08
			SMM	0.50
			TPM	0.27
	Jimma (n = 58)			
		Normal L-shaped	IAM	0.17
			SMM	0.05
			TPM	0.24

**Fig 4:**
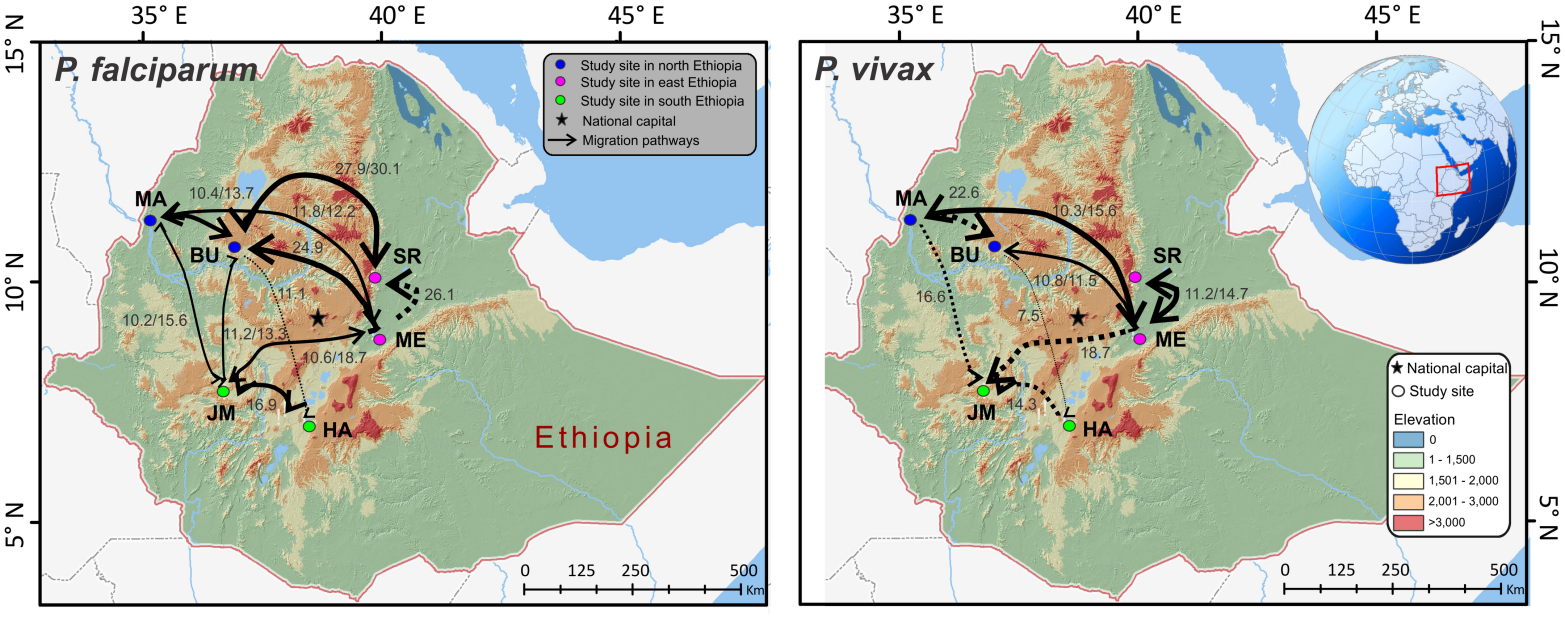
Migratory pathways and rates of *Plasmodium falciparum* and *P*. *vivax* among the study sites in Ethiopia. The intensity of gene flow is indicated by the thickness of migration paths. Solid lines denote bi-directional migration and dotted lines denote uni-directional migration. Only migration rate (*M*: immigration rate scaled by mutation rate) with a value of ≥10 are indicated on the maps. All values are presented in S6 Table.

Based on Migrate-N analyses, both *P*. *falciparum* and *P*. *vivax* showed a relatively small effective population size (Θ = 0.1–0.6 and 0.17–0.88, respectively; S5 Table), which suggested that the effect of drift was unequivocally as significant as migration. Given that most values of *M* (m/μ) were >1, the effect of migration (m) was larger than the effect of mutation (μ). For *P*. *falciparum*, the effective number of migrants per generation *N*_e_*m* ranged from 0.12–8.58. The greatest migration was observed between the north and east Ethiopia (e.g., from BU to SR: *M* 30.12 and *N*_e_*m* 8.58) and these north-east migrations were frequent and mostly bidirectional. While migrations between the north-east and south Ethiopia appeared to be less significant, these migrations in most cases were bidirectional (Fig 4). For *P*. *vivax*, the effective number of migrants per generation *N*_e_*m* ranged from 0.12–3.58. The greatest migration was between the northern populations (e.g., from MA to BU: *M* 22.58 and *N*_e_*m* 3.58) followed by the migration from the north and east to south Ethiopia (e.g., from ME to JM: *M* 18.77 and *N*_e_*m* 2.02; from MA to JM: *M* 16.60 and *N*_e_*m* 1.96). Interestingly, the migrations to the south were primarily unidirectional (Fig 4). BayesAss analyses supported the estimates of migration rates from Migrate-N. No significant correlations were found between migration rate and geographical distance in *P*. *falciparum* (*R*^2^ = 0.13, *P* = 0.09) and *P*. *vivax* (*R*^2^ = 0.02, *P* = 0.38; S2 Fig).

### Resistance gene marker polymorphisms

Among the 226 *P*. *falciparum* and 204 *P*. *vivax* samples, we successfully amplified and obtained complete resistance gene data in 199 (88%) and 185 (90%) of the samples (S7 Table). Samples with incomplete data were excluded in the analyses.

**Fig 5:**
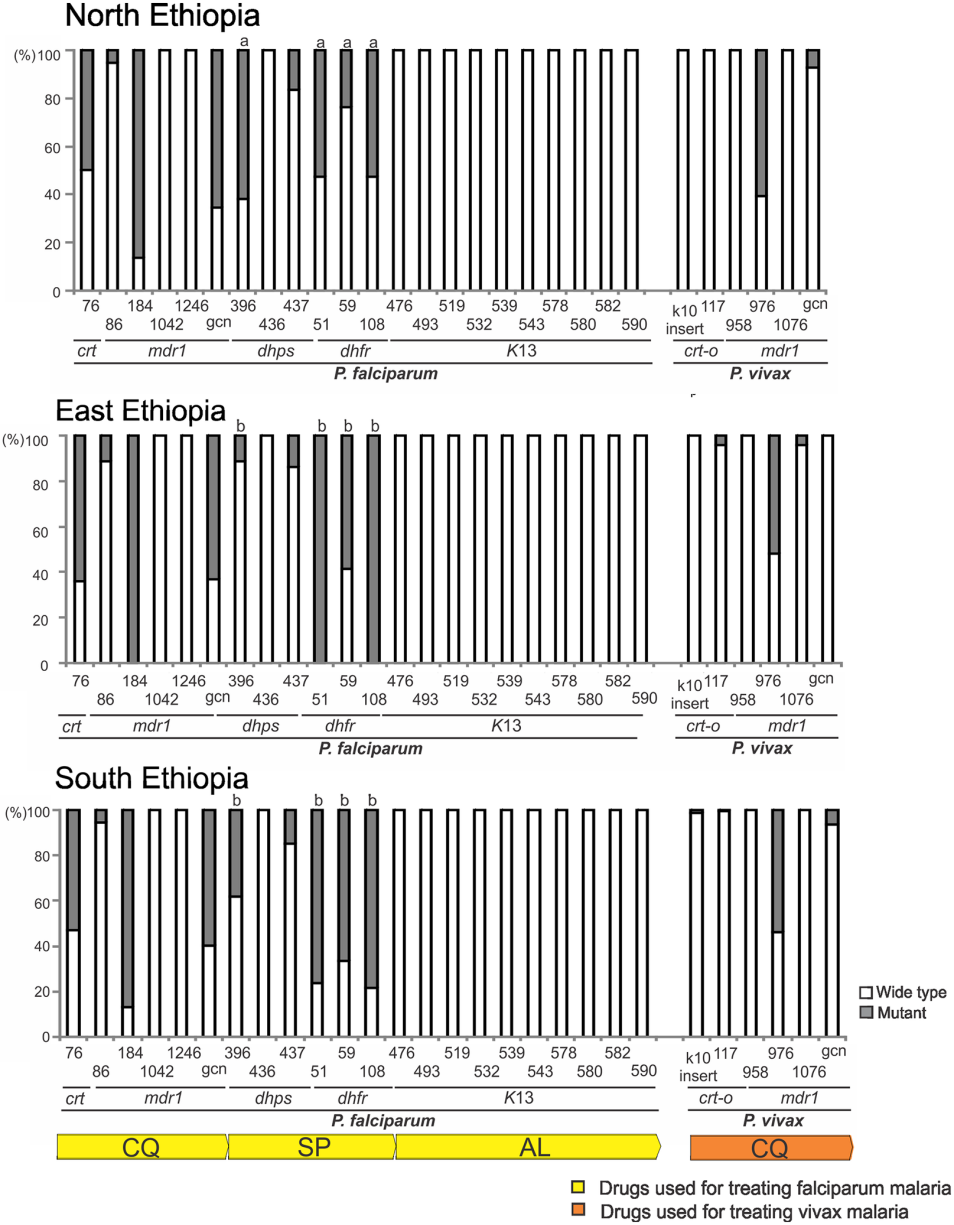
Frequency of mutations in gene codons related to antimalarial drug resistance (bottom bar) among *Plasmodium falciparum* and *P*. *vivax* samples from the north, east, and south Ethiopia. The proportion of wild type (white) and mutants (gray) for each codon position was indicated in each column of the respective resistant gene associated with the antimalarial drug (bottom bar). GCN denotes gene copy number of *pfmdr*1 and *pvmdr*1 (white: single copy; gray: ≥ two copies). Significant difference was detected in the mutation frequency of codons in *pfdhps* and *pfdhfr* among the study sites, as indicated by a small letter above the column.

*Plasmodium falciparum* samples from north, east and south Ethiopia all revealed a similar pattern of mutations in *pfcrt* and *pfmdr*1, the genes that associated with chloroquine resistance. In *pfcrt*, about 54–62% of the samples were shown with a mutant 76T genotype (north: 37/65 = 57%; east: 45/72 = 62.5%; south: 34/62 = 54.8%; Fig 5). While the majority of *P*. *falciparum* samples showed the wild type N86, N1042, and D1246 of *pfmdr*1, over 85% (north: 56/65 = 86%; east: 72/72 = 100%; south: 53/62 = 85.5%; Fig 5) of the samples had the mutant 184F genotype. Also, qPCR data indicated that over 60% of the samples had two or more copies of the *pfmdr*1 gene (north: 42/65 = 64.6%; east: 46/72 = 63.9%; south: 38/62 = 61.3%; Fig 5). The rate of mutations observed in *pfcrt* and *pfmdr*1 was not significantly different among populations.

By contrast, the pattern of mutation in genes *pfdhps* and *pfdhfr* that associated with SP resistance appeared to vary among geographical regions in Ethiopia (Fig 5). For instances, 62% (40/65) of *P*. *falciparum* in the northern populations had the mutant 396K of *pfdhps*, which was significantly higher than that in the eastern (8/72 = 11%) and southern populations (24/62 = 38%). While both the eastern and southern populations showed a preponderance of *pfdhfr* mutations in codons 51 (51I genotype; east: 72/72 = 100%; south: 48/62 = 77.4%), 59 (59R; east: 43/72 = 59.7%; south: 41/62 = 66%) and 108 (108N; east: 72/72 = 100%; south: 49/62 = 79%), the northern populations showed a significantly lower rate of mutations in these positions (51I: 34/65 = 52.3%; 59R: 15/65 = 23.2%; 108N: 34/65 = 52.3%; *P*<0.05). Amplification and sequencing of the entire *pfK13* indicated that this gene was highly conserved among all the *P*. *falciparum* samples. No polymorphisms were detected at the codon positions putatively related to artemisinin resistance (Fig 5; S7 Table).

For *P*. *vivax* samples from the north, east and south Ethiopia, all revealed a similar pattern of mutations in *pvcrt-o* and *pvmdr*1, the genes that associated with chloroquine resistance. Sequences of these two genes were highly conserved among the samples. Almost all had the wild type genotype except *pvmdr*1 codon 976 where over 50% of the samples had the mutant 976F genotype (north: 35/52 = 67.3%; east: 20/38 = 52.6%; south: 52/93 = 55.9%; Fig 5). qPCR data indicated that less than 4% of the samples had two or more copies of the *pvmdr*1 gene.

## Discussion

Ethiopia is a unique malaria endemic country in sub-Saharan Africa where *P*. *falciparum* and *P*. *vivax* coexist. The present study showed that both species revealed similar population structure. The northern and eastern populations of *P*. *falciparum* and *P*. *vivax* were genetically closely related and slightly distinct from the southern populations. While parasite gene flow was most frequent between the northern highlands and the highland-fringe areas along the Rift Valley, the southern basin populations were not excluded. We did not find a significant association between any landscape factors and population genetic structure. This result may be caused by the chosen metric for human movement (distance to roads), which does not fully capture the seasonal migration patterns that coincide with harvest season (i.e., September to November). The seasonal migration from highland to lowland areas for agricultural harvest appears to most closely reflect that of the observed gene flow patterns. Since the end of civil war in 1991, seasonal migration of Ethiopians from highland and highland-fringe areas to lowlands has increased due to the growth of large-scale agricultural development projects [67]. Sugarcane production and coffee plantations in the lowlands are important sources of employment and income to people who live in the highlands where agricultural activities are scarce. It is possible that agricultural employments in the eastern Rift Valley and southern basin areas elicit seasonal human migrations and consequently enable spread of *P*. *falciparum* and *P*. *vivax* across broad areas without landscape or distance barriers [68,69]. For example, the lowland districts of which populations can increase by 20–30% as a result of the arrival of tens of thousands of farmers during the harvest season [70,71]. Hence, in addition to maintaining control efforts in the community, seasonal migrant populations should not be ignored in order to effectively reduce malaria burden in Ethiopia. This may include close monitoring of malaria symptoms when seasonal migrant farmers return to their home village after the harvest season, as well as offering additional instructions and aids on prevention and prompt treatment of malaria.

At the gene level, *P*. *falciparum* and *P*. *vivax* revealed a comparable level of diversity within and among populations. The mean *H*_*e*_ in our *P*. *falciparum* populations (0.66) was similar to those reported in less endemic regions such as the Caribbean (*H*_*e*_ = 0.61) [72] and Indonesia (*H*_*e*_ = 0.53) [11]. Compared to endemic areas in South Pacific and Southeast Asia e.g., Papua New Guinea (*H*_*e*_ = 0.84) [12], central Vietnam (*H*_*e*_ = 0.88) [73], Cambodia (*H*_*e*_ = 0.84) [9] as well as a previous study in south-central Ethiopia (*H*_*e*_ = 0.82) [74], the mean *H*_*e*_ in our *P*. *vivax* populations (0.69) was slightly lower but similar to that in Asendabo (*H*_*e*_ = 0.70) [15], which is about 50km away from our study site Jimma in southern Ethiopia. The moderate and comparable genetic diversity observed in both species in Ethiopia contrasts with those reported in South Pacific and Southeast Asia where *P*. *vivax* showed a higher microsatellite diversity and less fragmented gene pool than the sympatric *P*. *falciparum* [9–12]. In countries or areas where malaria transmission is intense and stable, relapse could be common. This may, in turn, facilitate recombination and local spread of *P*. *vivax* leading to higher within-population diversity and lower among-population differentiation compared to *P*. *falciparum*. By contrast, our study sites in Ethiopia represent areas of low to moderate transmission settings where the relapse rate of *P*. *vivax* is largely unknown [35,75]. Malaria there displays a strong seasonal pattern with a lag time varying from a few weeks at the beginning of the rainy season to more than a month at the end of the rainy season [76]. The rainy season is relatively short in the highland and highland-fringe areas, and thus transmission season is usually short-lived [77]. These apparent seasonal and/or landscape differences not only influence the behavior and distribution of vector mosquitoes and the length of the parasite life cycle [78], but also the patterns of human movement and settlement that can in turn determine transmission dynamics of malaria. For instances, although *P*. *vivax* infections can occur periodically throughout the year, human migration is seasonal in Ethiopia and this could limit the spread of relapse *P*. *vivax*. Also, the long arid or semi-arid season particularly in the highlands may constrain gametocyte development of *P*. *vivax* or local transmission even when relapse occurs. A recent study in southern Ethiopia indicated an approximately 9.4% (ranged from 6.4–13.6% by sites) of *P*. *vivax* patients showed recurrent infection by day 28 [5,6]. However, because relapses can occur as early as 21 days following initial treatment, it is unclear how many of the recurrent cases were due to relapse. Given that our *P*. *falciparum* and *P*. *vivax* samples were collected at the same time during the peak transmission season, the lack of contrast between *P*. *falciparum* and *P*. *vivax* population structure and diversity may suggest similar demographic factors and a less significant impact of relapse. Future study should investigate the incidence of relapse among transmission settings and how such influences parasite population diversity.

The overall polyclonal infection rates observed in *P*. *falciparum* (8.8%) and *P*. *vivax* (4.3%) were low in our study area. The proportion of polyclonal *P*. *vivax* infections was similar to that reported in areas of low endemic setting such as Central China (2–19%) [79], but considerably lower than hypo-endemic areas such as Vietnam (71.4%; [80]), Sri Lanka (68%; [81]), Colombia (60–80%; [82]), and the Amazon Basin in Brazil (50%; [83]). Likewise, the polyclonal rate of *P*. *falciparum* was comparable to low transmission areas such as Haiti (12.9%) [72] and southern China (10–23%) [84] that are approaching elimination phase, but lower than endemic countries in West Africa such as Gambia and Senegal (36–50%) [85,86], East Africa such as Kenya (70–90%) [87], Papua New Guinea (39–45%) [88], and Southeast Asia such as Malaysia (65%) [89] and Cambodia (47%) [9].The higher polyclonality in *P*. *falciparum* than in *P*. *vivax* may imply a potentially large *P*. *falciparum* reservoir present in asymptomatic hosts that emerges during the transmission/rainy season [90]. By contrast, because *P*. *vivax* infections can occur periodically throughout the year in Ethiopia, our samples may reflect only a fraction of the existing *P*. *vivax* gene pool. Although relapse and early production and circulation of gametocytes in *P*. *vivax* infection can lead to increased opportunities for recombination, drug-sensitive clones (both blood-stage parasites or hypnozoites) could be eliminated during the non-transmission season by antimalarial treatment and resulted in reduced within-host diversity [8,82]. The lack of demographic data and drug use history of our patients limits our exploration of immunity and other factors on the observed MOI. It is important to note that microsatellites have the potential to underestimate polyclonality due to their lower sensitivity and specificity in detecting minority alleles compared to amplicon deep sequencing [91]. Despite the concern of relapse by *P*. *vivax*, the asymptomatic *P*. *falciparum* and *P*. *vivax* reservoirs that remain undetected during non-transmission season or in low endemic areas could pose a long-term impact on local transmission.

Ethiopia adopted AL as the first-line treatment for uncomplicated *P*. *falciparum* in 2004 in response to increased resistance to CQ and SP [2]. Unlike the Greater Mekong Subregion of SE Asia where delayed ACTs response has been reported, AL is shown to be highly effective in clearing parasite and fever within three days of drug treatment in Ethiopia [92]. The high efficacy of AL concords with our finding of predominantly wild type *pfK13* genotypes in *P*. *falciparum* from the northern, eastern, and southern populations. Although CQ and SP have not been used for *P*. *falciparum* treatment in the last decade, the high prevalence of mutations in *pfcrt* 76, *pfmdr1* 184, *pfdhps* 396, and *pfdhfr* 51 and 108 across Ethiopia suggested that strong selection may still exist possibly by the use of CQ for treating *P*. *vivax* malaria, as well as SP for intermittent preventive treatment (IPT) on pregnant women as part of the antimalarial schemes in sub-Saharan Africa [93]. It is concerning that the high prevalence of SP resistance mutations observed in the present study may indeed influence the outcome or effectiveness of IPT [94]. Another explanation for the high frequency of CQ and SP resistance mutations is the spread of resistance parasites from one population to another. Microsatellites indicated a substantial admixture of *P*. *falciparum* genotypes between north and east Ethiopia. It is possible that resistant parasites can spread via frequent human movements and become locally selected.

The observations of an alarming level of CQ resistance prevalence in Papua New Guinea [95], India [96], SE Asia [97,98], and South America [99] after a decade-application deepen the concern for the appearance of CQ resistance to *P*. *vivax* in Ethiopia. Recent reports of therapeutic failure of CQ (5.76–13%) [3–6] as well as high rates (9–32%) of recurrent infections subsequent to CQ usage in different parts of Ethiopia [6,100] have threatened the efficacy of *P*. *vivax* treatment. The highly conserved sequences of *pvcrt-o* as well as the predominantly wild type T958M, F1076L, and single copy of *pvmdr*1 suggest that these attributes may not be relevant to CQ resistance. By contrast, we detected a high prevalence of *pvmdr*1 976F mutation, which is considerably higher than that reported in India (22%) where the observed resistance genotypes were confirmed by *in vitro* drug sensitivity testing [101]. Thus, one possible explanation for the high 976F prevalence in our study area is that this mutation may associate with emerging CQ resistance. Such association merits further clinical observations and/or *in vitro* testing to confirm its functional significance. Moreover, it is yet unclear whether our *P*. *vivax* samples were relapse or recrudescent infections. In Cambodia, 89% of the *P*. *vivax* samples that were isolated from patients with recurrent/relapse infections within a 42-day follow-up had *pvmdr*1 976F mutation [102]. This reiterates the importance of distinguishing recrudescent from relapse infections in order to clarify the implications of the observed mutations and accurately elucidate resistance prevalence of *P*. *vivax*. Given that chloroquine monotherapy has been the recommended regimen for *P*. *vivax* malaria in Ethiopia for the past decades [2], selection as well as parasite gene flow may explain the emergence and spread of resistance genotypes across the country. Alternative *P*. *vivax* treatment regimes such as ACT or CQ in combination with primaquine are suggested to prolong the efficacy of CQ and prevent/reduce relapse in Ethiopia.

In summary, *P*. *falciparum* and *P*. *vivax* revealed moderate levels of genetic diversity and similar population structure in Ethiopia. Human migrations may promote parasite gene flow while environmental heterogeneity and geographical distance did not appear to be a major gene flow barrier. To effectively reduce malaria burden in Ethiopia, control efforts should focus on seasonal migrant populations. Unconstrained parasite gene flow may partly explain similar patterns of resistance marker prevalence across the country. Our findings are paramount to monitoring the emergence and spread of antimalarial drug resistance and offer evidence-based guidelines to existing treatment regimes.

## Supporting information

S1 Table Information of study location and sampling size. (DOCX)

S2 Table Microsatellite makers of *Plasmodium falciparum* and *P*. *vivax* used in the present study. (DOCX)

S3 Table Microsatellite genotypes of *Plasmodium falciparum* and *P*. *vivax* samples. (XLSX)

S4 Table Results of analyses of molecular variance (AMOVA) of *Plasmodium falciparum* and *P*. *vivax* samples partitioned by regions and populations. (DOCX)

S5 Table Membership coefficient (Q) of the most probable genetic clusters inferred by STRUCTURE for *Plasmodium falciparum* and *P*. *vivax*. (DOCX)

S6 Table Frequency of migration between sampling locations of *Plasmodium falciparum* and *P*. *vivax*. (DOCX)

S7 Table Resistance marker genotypes of *Plasmodium falciparum* and *P*. *vivax* samples. (XLSX)

S1 Fig A map of landscape connectivity between study sites based on Euclidean distance alone generated by Circuitscape. The scale of 1–10 (right) indicates the level of connectivity, e.g., areas withyellow represent the highest level of connectivity. Study sites were indicated by black dots.
(EPS)

S2 Fig Scatter plots showing the non-significant associations between migration rates estimated from BayesAss and geographical distances in *Plasmodium falciparum* and *P*. *vivax*. (EPS)
