# Supplementary material for: Transmission dynamics of co-endemic Plasmodium vivax and P. falciparum in Ethiopia and prevalence of antimalarial resistant genotypes
Source: PLoS Negl Trop Dis. 2017 Jul 26;11(7):e0005806. doi: 10.1371/journal.pntd.0005806 (PMC5546713; doi:10.1371/journal.pntd.0005806)
Supplement: S1 Table — (DOCX) [file pntd.0005806.s001.docx]

**S1 Table** Information of study location and sampling size.

| **Site** | **Label** | **Latitude, longitude** | ***P. falciparum* (*N*)** | ***P. vivax* (*N*)** |
| --- | --- | --- | --- | --- |
| **North Ethiopia** | | |  |  |
| Bure | BU | 10.70°N, 37.06°E | 42 | 39 |
| Mankush | MA | 11.27°N, 35.29°E | 36 | 19 |
| **East Ethiopia** | |  |  |  |
| Metehara | ME | 08.90°N, 39.92°E | 46 | 21 |
| Shewa Robit | SR | 10.00°N, 39.90°E | 33 | 21 |
| **South Ethiopia** | |  |  |  |
| Halaba | HA | 07.31°N, 38.09°E | 18 | 47 |
| Jimma | JM | 07.67°N, 36.83°E | 51 | 58 |
|  |  | **Total** | **226** | **205** |
