# Supplementary material for: Transmission dynamics of co-endemic Plasmodium vivax and P. falciparum in Ethiopia and prevalence of antimalarial resistant genotypes
Source: PLoS Negl Trop Dis. 2017 Jul 26;11(7):e0005806. doi: 10.1371/journal.pntd.0005806 (PMC5546713; doi:10.1371/journal.pntd.0005806)
Supplement: S2 Table — (DOCX) [file pntd.0005806.s002.docx]

**S2 Table** Microsatellite makers of *Plasmodium falciparum* and *P. vivax* used in the present study.

| ***P. falciparum*** | |  |  | ***P. vivax*** | |  |
| --- | --- | --- | --- | --- | --- | --- |
| Locus | Size range (bp) | Number of observed alleles |  | Locus | Size range (bp) | Number of observed alleles |
| TAA87 | 92-108 | 7 |  | MS1 | 224-246 | 10 |
| POLY2 | 95-137 | 10 |  | MS3 | 163-197 | 9 |
| PFPK2 | 161-198 | 15 |  | MS4 | 148-260 | 24 |
| 9735 | 105-143 | 10 |  | MS5 | 161-187 | 10 |
| TAA109 | 155-191 | 11 |  | MS8 | 201-269 | 13 |
| TAA42 | 176-200 | 8 |  | MS9 | 120-168 | 15 |
| TAA81 | 110-164 | 12 |  | MS10 | 177-232 | 22 |
| PE87a | 119-164 | 13 |  | MS12 | 203-237 | 13 |
| POLYα | 168-241 | 19 |  | MS15 | 216-268 | 14 |
| PFG377 | 85-101 | 5 |  | MS16 | 190-293 | 19 |
| TA80 | 101-178 | 6 |  | MS20 | 153-251 | 24 |
| TA116 | 154-190 | 13 |  | Pv3.27 | 103-190 | 20 |
| TA60 | 174-211 | 12 |  | Pv1.501 | 101-170 | 21 |
