# Supplementary material for: Transmission dynamics of co-endemic Plasmodium vivax and P. falciparum in Ethiopia and prevalence of antimalarial resistant genotypes
Source: PLoS Negl Trop Dis. 2017 Jul 26;11(7):e0005806. doi: 10.1371/journal.pntd.0005806 (PMC5546713; doi:10.1371/journal.pntd.0005806)
Supplement: S4 Table — (DOCX) [file pntd.0005806.s004.docx]

**S4 Table** Results of analyses of molecular variance (AMOVA) of *Plasmodium falciparum* and *P. vivax* samples partitioned by regions and populations.

| **Samples** | | **Source of variation** | **Percentage of variation** | ***P*-value** |
| --- | --- | --- | --- | --- |
| ***P. falciparum*** | |  |  |  |
|  |  | Among regions | 13 | 0.001 |
|  |  | Among populations | 3 | 0.053 |
|  |  | Within populations | 84 | 0.001 |
| ***P. vivax*** | |  |  |  |
|  |  | Among regions | 8 | 0.001 |
|  |  | Among populations | 7 | 0.001 |
|  |  | Within populations | 85 | 0.001 |
