# Supplementary material for: Transmission dynamics of co-endemic Plasmodium vivax and P. falciparum in Ethiopia and prevalence of antimalarial resistant genotypes
Source: PLoS Negl Trop Dis. 2017 Jul 26;11(7):e0005806. doi: 10.1371/journal.pntd.0005806 (PMC5546713; doi:10.1371/journal.pntd.0005806)
Supplement: S5 Table — (DOCX) [file pntd.0005806.s005.docx]

**S5 Table** Membership coefficient (Q) of the most probable genetic clusters inferred by STRUCTURE for *Plasmodium falciparum* and *P. vivax*. Values of Q>0.4 are in bold and represents a dominant genetic cluster.

| ***P. falcipaum*** | |  |  |  |  | ***P. vivax*** |  |  |  |
| --- | --- | --- | --- | --- | --- | --- | --- | --- | --- |
| Site | K1 | K2 | K3 | *N* |  | Site | K1 | K2 | *N* |
| Mankush | 0.263 | 0.075 | **0.661** | 36 |  | Mankush | **0.937** | 0.063 | 19 |
| Bure | 0.353 | **0.405** | 0.242 | 42 |  | Bure | **0.936** | 0.064 | 39 |
| Shewa Robit | **0.635** | 0.14 | 0.226 | 33 |  | Shewa Robit | **0.954** | 0.046 | 21 |
| Metahara | 0.342 | **0.461** | 0.197 | 46 |  | Metahara | **0.898** | 0.102 | 21 |
| Jimma | 0.178 | 0.304 | **0.519** | 51 |  | Jimma | **0.715** | 0.285 | 58 |
| Halaba | 0.071 | **0.568** | 0.36 | 18 |  | Halaba | 0.356 | **0.644** | 47 |
