# Supplementary material for: Transmission dynamics of co-endemic Plasmodium vivax and P. falciparum in Ethiopia and prevalence of antimalarial resistant genotypes
Source: PLoS Negl Trop Dis. 2017 Jul 26;11(7):e0005806. doi: 10.1371/journal.pntd.0005806 (PMC5546713; doi:10.1371/journal.pntd.0005806)
Supplement: S6 Table — (DOCX) [file pntd.0005806.s006.docx]

**S6 Table** Frequency of migration between sampling locations of *Plasmodium falciparum* and *P. vivax*. Values in parentheses indicated standard deviation. Θ: effective population size scaled by mutation rate per generation and site (4N_e_μ);*M*: migration rate by mutation rate (m/μ). Cells in blue represent populations in North Ethiopia; pink for East Ethiopia; and green for South Ethiopia.

***P. falciparum***

| **Source population** |  | ***M* estimates for sink populations** | | | | | |
| --- | --- | --- | --- | --- | --- | --- | --- |
|  | Θ | BU | MA | ME | SR | HA | JM |
| BU | 0.57  (0.51-0.64) | - | **10.43**  (8.23-13.0) | 6.66  (4.83-8.89) | **30.12**  (26.22-34.4) | **11.08**  (7.72-13.31) | **13.34**  (11.11-15.85) |
| MA | 0.38  (0.35-0.42) | **13.73**  (11.29-16.50) | - | 12.15  (9.62-15.08) | 4.22  (2.87-5.94) | 3.11  (1.51-5.57) | **15.56**  (12.81-18.67) |
| ME | 0.10  (0.09-0.11) | **24.94**  (21.53-28.64) | **11.85**  (9.47-14.60) | - | **26.10**  (22.49-30.09) | 8.36  (5.49-12.09) | **18.72**  (16.04-21.70) |
| SR | 0.38  (0.34-0.42) | **27.96**  (24.40-31.85) | 9.31  (7.23-11.76) | 7.56  (5.60-9.97) | - | 6.81  (4.25-10.21) | 7.70  (6.01-9.65) |
| HA | 0.15  (0.13-0.18) | 7.30  (5.57-9.36) | 2.54  (1.54-3.91) | 1.66  (0.84-2.88) | 4.55  (3.15-6.32) | - | 7.35  (5.72-9.26) |
| JM | 0.60  (0.55-0.66) | **11.02**  (8.85-13.53) | **10.18**  (8.25-12.39) | 10.59  (8.24-13.35) | 7.50  (5.82-9.95) | **16.95**  (12.67-22.12) | - |

***P. vivax***

| **Source population** |  | ***M* estimates for sink populations** | | | | | | |  | | |
| --- | --- | --- | --- | --- | --- | --- | --- | --- | --- | --- | --- |
|  | Θ | | MA | BU | ME | SR | HA | JM |  |  |  |
| MA | 0.22  (0.19-0.25) | | - | **22.58**  (18.12-26.13) | **15.62**  (11.9-19.5) | 5.09  (3.3-7.42) | 6.38  (3.9-9.69) | **16.60**  (14.77-19.31) |  |  |  |
| BU | 0.80  (0.72-0.93) | | 1.21  (0.41-2.71) | - | **11.48**  (7.01-14.5) | 2.23  (1.14-3.88) | 7.49  (4.77-12.57) | 4.74  (3.63-6.05) |  |  |  |
| ME | 0.17  (0.16-0.28) | | **10.32**  (6.17-14.02) | **10.79**  (8.8-13.79) | - | **11.15**  (7.75-16.69) | 4.77  (2.6-7.74) | **18.77**  (16.14-22.43) |  |  |  |
| SR | 0.18  (0.16-0.2) | | 2.65  (1.45-4.38) | 4.92  (3.33-6.94) | **14.74**  (12.1-17.78) | - | 0.77  (0.14-2.22) | 6.75  (5.22-8.54) |  |  |  |
| HA | 0.52  (0.22-1.78) | | 0.46  (0.03-1.38) | 2.56  (1.34-3.73) | 1.38  (0.58-2.67) | 1.55  (0.52-3.45) | - | **14.33**  (12.31-16.54) |  |  |  |
| JM | 0.88  (0.82-1.1) | | 3.77  (2.14-6.05) | 8.61  (6.18-11.28) | 4.44  (3.06-6.19) | 4.23  (2.62-6.38) | 5.08  (3.47-7.13) | - |  |  |  |
